# The relationship between metabolite mediated immune regulatory imbalance and the occurrence of malignant tumors of bone and articular cartilage: a Mendelian randomization study

**DOI:** 10.3389/fimmu.2024.1433219

**Published:** 2024-08-09

**Authors:** Kehan Long, Ao Gong, Tengfei Zheng, Shoushen Liu, Zhendong Ying, Cong Xiao

**Affiliations:** ^1^ Department of Orthopedics, The Third Hospital of Mianyang· Sichuan Mental Health Center, Mianyang, China; ^2^ Department of Orthopedics, Second Clinical Medical College of Shandong University of Traditional Chinese Medicine, Jinan, Shandong, China; ^3^ Department of Orthopedics, Shandong First Medical University, Jinan, Shandong, China

**Keywords:** causal relationships, immune cell features, Mendelian randomization, malignant tumors of bone and articular cartilage, metabolites, mediation analysis

## Abstract

**Background:**

This study aims to assess the causal relationship between immune cell characteristics and malignant tumors of bone and articular cartilage, focusing on the mediating role of metabolites. Using Mendelian randomization, we evaluated these relationships based on genetic variations to identify potential biomarkers and therapeutic targets.

**Methods:**

A two-sample Mendelian randomization analysis was conducted using GWAS data for immune cell features and 1,400 metabolites to investigate direct and mediating effects. Effective instrumental variables (IVs) were selected, and statistical analyses—including inverse variance weighting (IVW), weighted median, and mode-based methods—were performed using R software. This approach enabled the assessment of direct causal relationships as well as the potential mediating role of metabolites in the association between immune cell features and malignancies.

**Results:**

Significant causal relationships were identified between 26 immune phenotypes and the risk of malignant tumors of bone and articular cartilage. Notably, the HLA DR+ NK cell phenotype SSC-A showed a positive correlation with the risk of these malignancies. Further analysis revealed causal relationships with 67 metabolites, 38 of which were positively correlated and 29 negatively correlated. Mediation analysis highlighted the role of immune surveillance and metabolic dysregulation in tumor development, as evidenced by the association between the immune phenotype SSC-A on HLA DR+ NK cells and the metabolite 5-hydroxyhexanoate.

**Conclusion:**

The findings suggest significant causal relationships between immune phenotypes and malignant tumors of bone and articular cartilage, with metabolites potentially mediating these relationships. These insights lay the groundwork for further research and could contribute to the development of new biomarkers and treatment strategies.

## 1 Introduction

Malignant tumors of bone and articular cartilage represent relatively rare yet highly aggressive cancers characterized by poor prognosis, limited treatment options, and low survival rates (1). Primary bone cancers constitute approximately 0.2% of all malignancies, with osteosarcoma and Ewing sarcoma being the most common types in children and adolescents. These tumors often metastasize to the lungs and other bones, leading to poor clinical outcomes (2–4). Chondrosarcomas represent the most prevalent primary malignant bone tumor in adults, while other cartilage malignancies like clear cell chondrosarcoma and mesenchymal chondrosarcoma are exceptionally rare (5). The complex anatomy and distinctive biomechanical microenvironments of bone and cartilage impose unique selective pressures during oncogenesis and progression. Additionally, the low vascularity and abundance of extracellular matrix in cartilage pose therapeutic delivery challenges (6). Current standard care heavily relies on surgical resection combined with neoadjuvant or adjuvant chemotherapy and radiation. However, chemoresistance and detrimental impacts on bone growth in young patients limit these conventional modalities (7, 8). Accumulating evidence indicates that the dynamic interplay between immune surveillance mechanisms and metabolic pathways may critically influence tumorigenesis and progression (9). Specifically, recent omics-based studies reveal that immune cell phenotypes and metabolic profiles could provide valuable biomarkers for risk assessment and targeted intervention. However, conventional observational analytic approaches are intrinsically constrained in their ability to disentangle causal relationships from confounding factors and reverse causation (10). Mendelian randomization (MR) overcomes this barrier by utilizing genetic variants as instrumental variables (IVs) to probe causal associations free of biases.

This study implements a comprehensive MR framework integrating two-sample analyses to systematically evaluate causal effects of a broad spectrum of immune traits and metabolites on malignant bone and cartilage tumors. A total of 731 immune phenotypes were examined, encompassing counts, surface markers, and morphological features for diverse cell types including B cells, dendritic cells, T cells, natural killer cells, monocytes, and regulatory T cells (11). Additionally, 1,400 blood metabolites representing potential intermediary endpoints were analyzed to map dysregulated metabolic pathways that may drive tumorigenesis. This dual-sample MR design leverages summary-level genome-wide association study (GWAS) data as IVs for the exposure (immune traits) and outcome (tumor risk). By exploiting the random assortment of alleles during meiosis, MR minimizes confounding and establishes temporality (12).

Rigorous statistical techniques were implemented to derive robust causal estimates, including inverse variance weighted (IVW), MR Egger, and weighted median methods (13). Complementary sensitivity analyses account for horizontal pleiotropy, and Steiger filtering precludes reverse causation (14). Moreover, mediation analysis quantifies direct versus indirect effects, delineating mechanisms whereby metabolites may mediate impacts of aberrant immune activity on malignant progression.

This comprehensive investigation aims to elucidate causal pathways driving rare yet aggressive bone and cartilage cancers. The findings promise to uncover novel biomarkers for early detection and prognosis. By spotlighting critical immune phenotypes and metabolic profiles, this MR study could inform the development of personalized risk prediction models to guide targeted screening and prevention strategies. The mediation analysis specifically assesses metabolic factors representing potential key mechanistic links between immune dysregulation and uncontrolled tumor growth that may constitute promising drug targets.

## 2 Materials and methods

### 2.1 Study design

**Figure 1:**
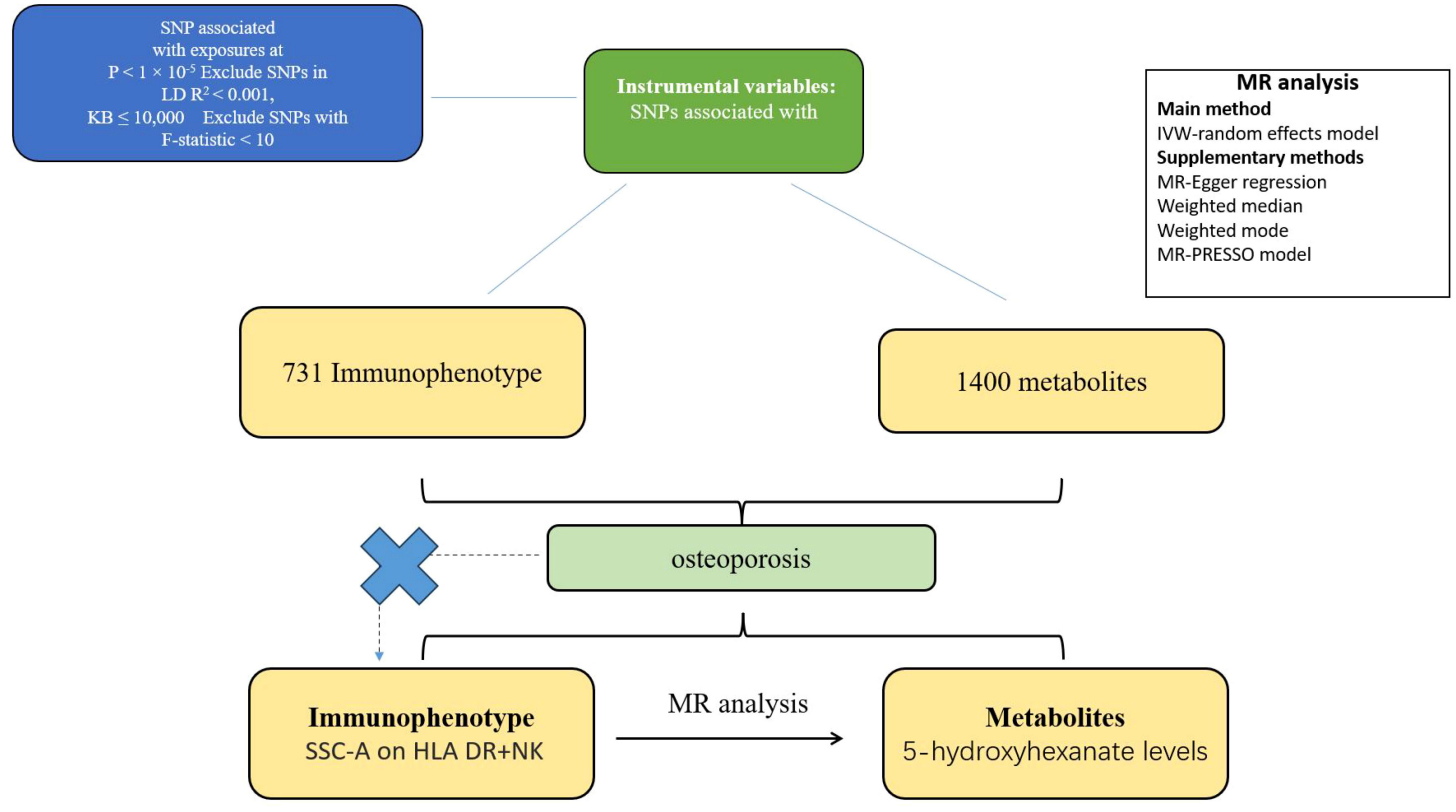
Flow chart of two sample and mediator Mendelian randomization analysis. This flowchart outlines the Mendelian randomization method for two samples to investigate the causal effect of immune cell phenotype on the risk of metabolic product mediated bone and articular cartilage malignancy. The selection of SNP is based on the correlation with exposure level (*P*<1x10^-5^), LD threshold (R^2^<0.001), and F-statistic (>10), ensuring the robustness of instrumental variables. This analysis confirms the absence of confounding factors and indirect pathways, following the Mendelian randomization hypothesis. And evaluate the causal relationship between immune phenotype and metabolic product mediated malignant tumors of bone and articular cartilage.

Based on dual sample MR analysis, we evaluated the mediating causal relationship between immune cells and malignant tumors of bone and articular cartilage through the influence of metabolites. MR uses genetic variation to represent risk factors, and effective instrumental variables (IVs) in causal reasoning, as depicted in **Figure 1**, must satisfy three key assumptions (1): genetic variation is directly related to exposure (2); genetic variation is not associated with potential confounding factors between exposure and outcome (3); genetic variation does not affect the results through pathways other than exposure (15–17). **Figure 1** intuitively illustrates the causal relationship with metabolites acting as mediators between immune cells and malignant tumors of bone and articular cartilage. This study is reported following the Strengthening the Reporting of Observational Studies in Epidemiology Using Mendelian Randomization guidelines (STROBE-MR, S1 Checklist) (18).

### 2.2 GWAS data for all immune features and 1400 metabolites

The comprehensive GWAS summary data for each immune feature can be publicly accessed from the GWAS catalog under registration numbers GCST90001391 to GCST90002121. This includes a total of 731 immune phenotypes, comprising absolute cell counts (AC) (n=118), median fluorescence intensity reflecting surface antigen levels (MFI) (n=389), morphological parameters (MP) (n=32), and relative cell counts (RC) (n=192). Specifically, MFI, AC, and RC features encompass B cells, common dendritic cells (CDCs), mature T cells, monocytes, bone marrow cells, TBNK (T cells, B cells, natural killer cells), and Treg cells, while MP features include CDC and TBNK cells (19). The initial GWAS for immune features utilized data from 3,757 individuals of European descent with no overlapping data. Approximately 22 million high-density array genotypes of SNPs were computed using a reference panel based on the Sardinian population sequence, and correlations were examined after adjusting for covariates (i.e., gender and age) (20). Aggregate GWAS statistics for each metabolite are publicly available from the GWAS catalog under registration numbers GCST90199621 to GCST90201020. This large-scale GWAS study includes 1,091 metabolites and 309 metabolite ratios from 8,299 individuals in the Canadian Longitudinal Aging Study (CLSA) cohort (21). Data on malignant tumors of bone and articular cartilage come from R10, including 224 cases and 314,193 controls.

### 2.3 Selection of instrumental variables

Due to the direct correlation between genetic variation and exposure, the significance level of IVs for each immune feature is set at 1 × 10^-5^. Similarly, the significance level for metabolites in IVs is also set at 1 × 10^-5^. To obtain the IVs of independent sites, we used the “TwoSampleMR” package with a linkage disequilibrium (LD) threshold set to R^2^ < 0.001 and an aggregation distance of 10,000 kb (22). For malignant tumors of bone and articular cartilage, we adjusted the significance level to 1 × 10^-5^, which is commonly used to represent genome-wide significance in GWAS, with an LD threshold of R^2^ < 0.001 and an aggregation distance of 10,000 kb. We calculated the F value of each SNP and excluded SNPs with F < 10, as well as palindrome SNPs (23).

### 2.4 Statistical analysis

A double sample Mendelian randomization analysis of positive metabolites was performed on immune cell phenotypes using R software, version 4.2.1, which is a widely used statistical calculation and graphical environment (http://www.Rproject.org). The “TwoSampleMR” package (version 0.5.7) in the R software environment facilitated these analyses. This software package is specifically designed for MR analysis, providing tools for estimating, testing, and conducting sensitivity analyses of causal effects (22). The IVW method, a standard approach in MR, combines Wald estimates from multiple genetic variations (the ratio of SNP result associations to SNP exposure associations) and is weighted by the inverse variance associated with each SNP result. Weighted median and mode-based methods serve as supplementary approaches, providing robust causal estimates even if certain instrumental variables are invalid, as long as specific assumptions are met (24, 25). These analyses are supported by rigorous sensitivity tests, including Cochran’s Q-test, to examine heterogeneity between instrumental variables. This thorough statistical evaluation ensures that the results are as reliable and accurate as possible given the data.

### 2.5 Mendelian mediation analysis

Firstly, we analyzed the causal relationship between immune characteristics and malignant tumors of bone and articular cartilage, eliminating the interference of reverse causal relationships in this step. We then further analyzed the causal effects of metabolites on malignant tumors of bone and articular cartilage. Next, we selected the immune feature with the lowest *P*-value in the causal relationship between malignant tumors of bone and articular cartilage as the exposure, analyzed it with positive metabolites, and finally determined the mediating factor. The overall impact of immune characteristics on malignant tumors of bone and articular cartilage can be decomposed into direct and indirect effects. The indirect effect is calculated as the product of the causal relationship between immune characteristics and metabolites (β value) and the causal relationship between metabolites and malignant tumors of bone and articular cartilage (β value) (26). The direct effect is equal to the total effect minus the indirect effect. The mediation ratio is calculated as the mediation effect divided by the total effect (27).

## 3 Results

### 3.1 The causal relationship between immune phenotype and malignant tumors of bone and articular cartilage

**Figure 2:**
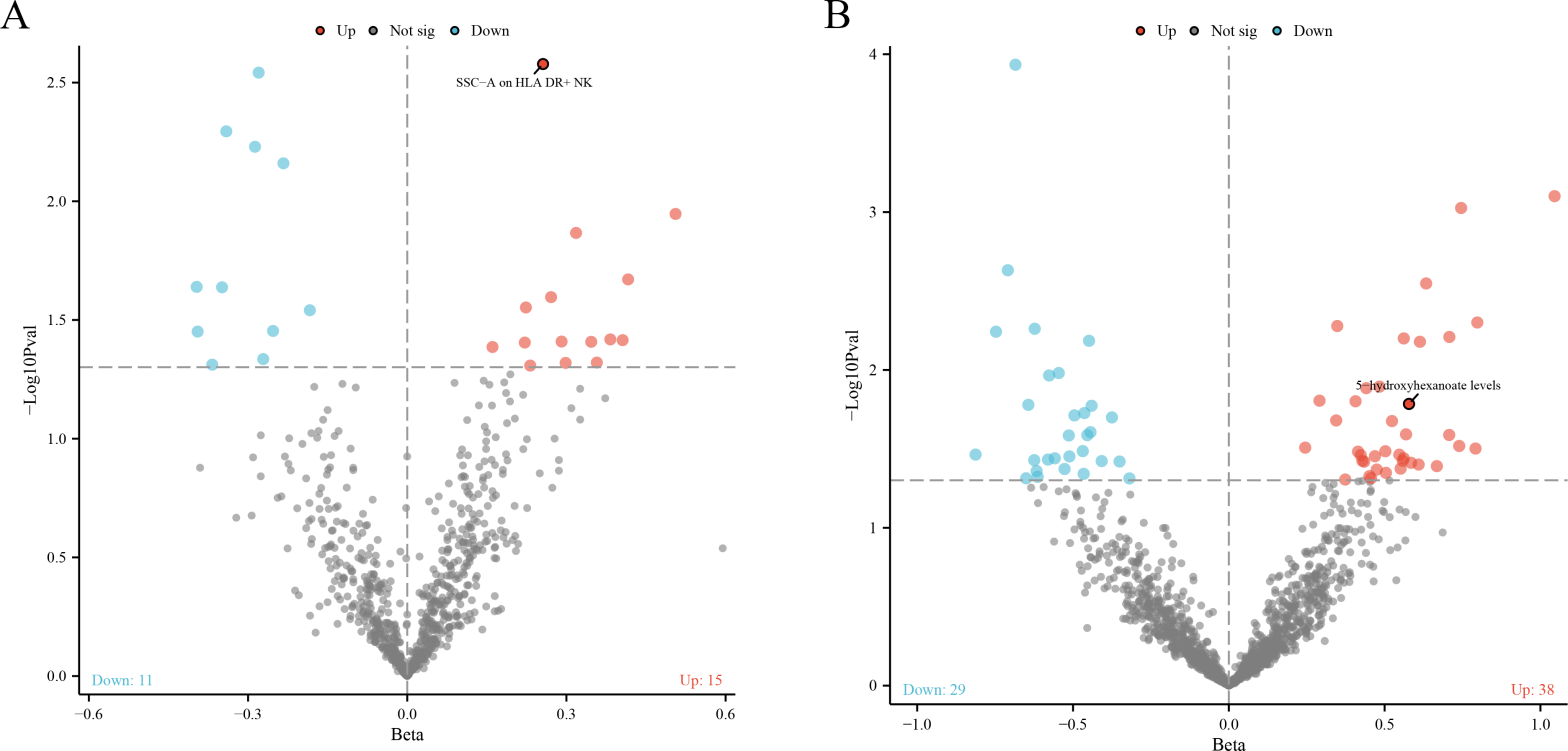
**(A)** The volcano plot shows a significant causal relationship between the 731 immune phenotype and the risk of malignant tumors in bone and articular cartilage. Each fragment corresponds to a single immune phenotype, and the phenotype feature SSC-A on HLA DR+NK cells is highlighted, indicating a positive correlation with tumor risk. The graph aims to convey the extensive analysis conducted, including 26 immune phenotypes that demonstrate a significant impact on tumor risk. **(B)** The volcano plot shows the causal relationship between 1400 metabolites and malignant tumors of bone and articular cartilage. This panel displays metabolites that are causally associated with malignant tumors. According to the IVW method, 38 metabolites are positively correlated and 29 metabolites are negatively correlated.

**Figure 3:**
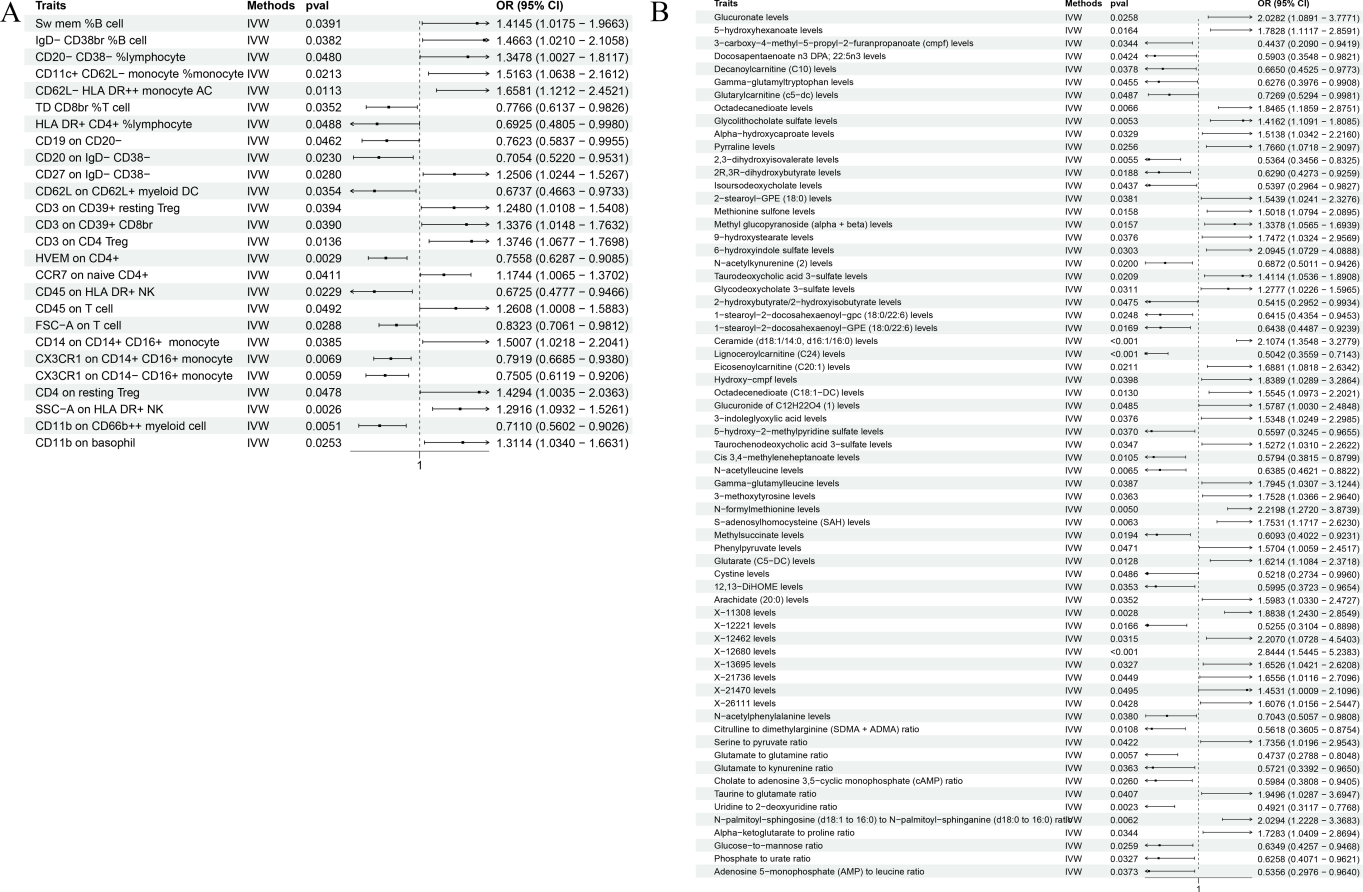
The forest plot shows the correlation between metabolic products and immune phenotype with the risk of malignant tumors in bone and articular cartilage. **(A)** The causal relationship between 26 positive immune phenotypes and malignant tumors of bone and joint cartilage; **(B)** The causal relationship between 67 positive metabolites and malignant tumors of bone and articular cartilage.

In our robust Mendelian randomization analysis using the Inverse Variance Weighted (IVW) method, we identified a significant interplay between immune phenotypes and the risk of malignant tumors in bone and articular cartilage. This comprehensive study revealed 26 immune phenotypes with a significant causal effect on these malignancies at a significance level of 0.05, as detailed in **Figures 2A**, **3A**. Notably, the phenotype SSC-A on HLA DR+NK emerged as a prominent factor, exhibiting a positive correlation with these malignant tumors. The association’s strength was underscored by a compelling *P*-value of 0.002, indicating a less than 0.2% probability that this correlation is due to chance. Furthermore, the effect size (β) of 0.255 and the odds ratio (OR) of 1.291, with a 95% confidence interval ranging from 1.093~1.526, bolster the evidence for its biological relevance, as depicted in **Figure 3A**. However, our analysis also indicates that there is no direct causal relationship between bone tumors and articular cartilage tumors, and SSC-A on HLA DR+NK (P=0.379). To further ensure the robustness of our MR results and to address the issue of possible cross-sectional heterogeneity, we performed an Egger intercept analysis and collated the results in **Supplementary Table 1**.

### 3.2 The causal relationship between metabolites and malignant tumors of bone and articular cartilage

Our comprehensive statistical analysis using the Inverse Variance Weighted (IVW) method, with a predetermined alpha significance level of 0.05, has unveiled a complex network of associations between metabolites and malignant tumors of bone and articular cartilage. Specifically, our study has identified 67 metabolites that display a causal relationship with these malignancies. Delving into the nature of these relationships, 38 metabolites were found to positively influence the development of these tumors, suggesting that their increased levels could be predictive of a higher risk for these cancer types (as show in **Supplementary Table 2**). Conversely, 29 metabolites were negatively associated, indicating a potential protective effect or lower risk of tumor development when these metabolite levels are elevated (as show in **Supplementary Table 3**). These findings, graphically represented in **Figures 2B**, **3B**, provide critical insights into the metabolic pathways that may contribute to the pathogenesis of bone and articular cartilage tumors. To enhance the reliability of our MR findings and tackle the potential issue of cross-sectional heterogeneity, we conducted an Egger intercept analysis and compiled the outcomes in **Supplementary Table 4**. Of the 67 metabolites identified, we found that 5 data had horizontal pleiotropy, so they should have been analyzed using weighted medians as the primary method. The results were Isoursodeoxycholate levels (P=0.035, OR=0.444, 95%CI=0.209 ~ 0.943), X-13695 levels (P=0.024, OR=2.073, 95%CI=1.099 ~ 3.911) and N-palmitoyl- sphingosine (d18:1 ~ 16:0) to N-palmitoyl-sphinganine (d18:0 ~ 16:0) ratio (P=0.014, OR=2.364, 95%CI=1.194~4.682), the remaining two 9-hydroxystearate levels (P=0.126, OR=1.760, 95%CI=0.854~3.630) and S-adenosylhomocysteine (SAH) levels (P=0.251, OR=1.410, 95%CI=0.784~2.536) were not statistically significant after weighted median analysis (P>0.05).

### 3.3 The causal relationship between immune characteristics and 67 metabolites

In our focused Mendelian randomization analysis, we meticulously selected the immune phenotype SSC-A on HLA DR+NK and the top 67 metabolites based on their strong causal linkage with malignant tumors of bone and articular cartilage. The analysis yielded significant results, particularly highlighting the relationship between the immune feature SSC-A on HLA DR+NK and the metabolite 5-hydroxyhexanoate. Statistical significance was confirmed with a *P*-value of 0.017, indicating a 1.032-fold increase in odds ratio (OR) for the association, with a tight 95% confidence interval ranging from 1.005~1.060. These detailed results are presented in **Table 1**, elucidating the potential mechanistic link between immune surveillance and metabolic dysregulation in the context of these tumors. To additionally safeguard the integrity of our MR findings and confront the challenge of potential cross-sectional variability, we executed an Egger intercept analysis and assembled the findings in **Supplementary Table 5**.

**Table 1:** Causal relationship between immune phenotype SSC-A on HLA DR+NK and metabolite 5-hydroxyhexanate levels.

method	nsnp	pval	Beta	or	or_lci95	or_uci95
MR Egger	28	0.046323	0.038024	1.038756	1.002407	1.076424
Weighted median	28	0.025105	0.045366	1.046411	1.005683	1.088788
Inverse variance weighted	28	0.017856	0.032207	1.032731	1.005571	1.060625
Simple mode	28	0.547036	0.020754	1.020971	0.955096	1.091388

### 3.4 Mendelian mediation analysis

**Figure 4:**
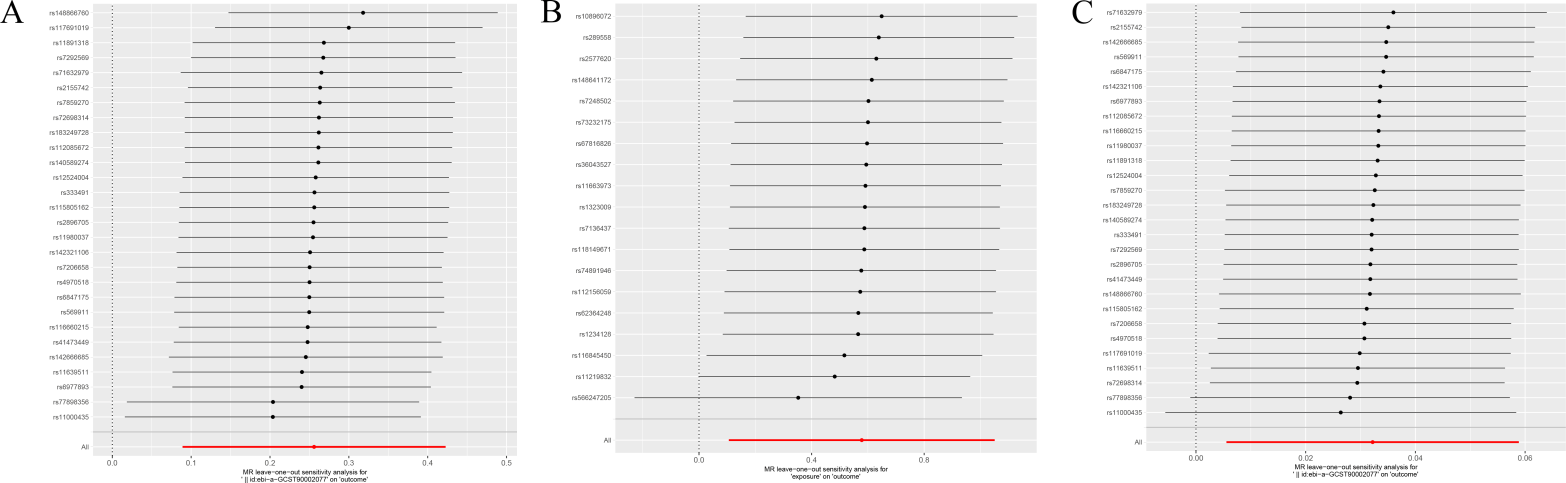
**(A)** Displays the sensitivity analysis of the association between SSC-A on HLA DR+NK and malignant tumors of bone and articular cartilage. **(B)** Illustrates the sensitivity analysis for the relationship between 5-hydroxyhexanoate levels and malignant tumors of bone and articular cartilage. **(C)** Presents the sensitivity analysis for the correlation between SSC-A on HLA DR+NK and 5-hydroxyhexanoate levels. Each panel depicts the influence of individual data points on the overall estimate when sequentially omitted, ensuring the robustness of the associations analyzed.

In our quantitative mediation analysis, we discerned that the immune phenotype SSC-A on HLA DR+NK contributes significantly to the etiology of malignant tumors of bone and articular cartilage. The calculated indirect effect of 0.018 and the direct effect of 0.237, derived from the total effect of 0.255, reveal the dual roles of immune surveillance and metabolic factors in tumorigenesis. Specifically, the metabolite 5-hydroxyhexanoate was found to mediate this process with a mediation ratio of 7.2%, underscoring its role in the biological pathway. The results in each direction were analyzed for sensitivity by the leave-one-out method, which showed no outliers (**Figure 4**).

## 4 Discussion

In this study, we implemented a comprehensive Mendelian randomization approach integrating genetic instruments for immune phenotypes, metabolites, and malignancies of bone and cartilage. Our robust statistical analysis uncovered significant causal relationships and highlighted potential mechanistic links mediated through metabolic dysregulation. These novel findings elucidate the complex interplay between immune surveillance and metabolic pathways in the pathogenesis of rare yet aggressive bone and cartilage tumors.

Through our wide-ranging Mendelian randomization screen encompassing over 731 immune cell types, we identified 26 specific immune cell phenotypes that demonstrated statistically significant causal impacts on risk for bone and cartilage malignancies after stringent multiple testing correction. Among these prioritized cell types, the cell surface marker SSC-A on HLA-DR+ NK cells emerged as a particularly compelling finding, exhibiting a strong positive correlation with increased tumor risk. HLA-DR+ NK cells represent an activated NK cell subset endowed with enhanced effector functions but also susceptible to hyperactivity that could precipitate break down of self-tolerance (28). Our study provides the first evidence that dysregulated granularity as assessed specifically by SSC-A, a measure of granularity reflecting lysosome content, may play a critical and previously unrecognized role in instigating the pathological transformation of HLA-DR+ NK cells towards generators of chronic inflammation that ultimately foster tumorigenesis (29).

Elevated SSC-A levels likely reflect heightened lysosomal activation and secretory function, leading to overproduction of inflammatory cytokines and uncontrolled NK cell cytotoxicity that could damage healthy cells and tissues (30, 31). This nominates the SSC-A phenotype on HLA-DR+ NK cells as a potential immunological prognostic biomarker and driver of bone/cartilage cancers. Monitoring SSC-A levels via high dimensional flow cytometry of patient blood samples could enable earlier detection of aberrant HLA-DR+ NK activation before full malignancy develops, allowing for preventative interventions in high-risk groups (32). Additionally, directly targeting the molecular pathways involved in HLA-DR+ NK cell maturation and functional regulation using novel immunotherapeutics represents a promising strategy to restrain their dysregulation and curb downstream inflammatory and oxidative stress known to enable tumor progression (33).

Our multi-level analysis expands in scope beyond immune phenotypes to uncover 67 key metabolites with significant causal associations with bone and cartilage tumor risk after adjusting for multiple comparisons. Rather than mere correlational bystanders, these prioritized metabolites are functionally relevant players in cancer-associated pathways related to inflammation, proliferation, redox homeostasis, and central carbon metabolism. A prime example is adenine, whose elevated levels were positively correlated with heightened malignancy risk (34, 35). Adenine is far more than an inert metabolic substrate; it is an active signaling molecule that participates in pro-inflammatory purinergic signaling cascades known to exacerbate metastasis and angiogenesis (36). This highlights the complex and multifaceted roles of purines like adenine in enabling key hallmarks of tumor initiation and progression.

In contrast, elevated levels of the amino acid histidine were found to have protective influences on malignancy risk, likely attributable to its antioxidant properties that attenuate damaging oxidative stress (37). This underscores how the context-dependent dual nature of amino acids in different cancer biology settings produces divergent functional impacts. Additionally, itaconate, an immunometabolite produced as a byproduct of the activation of tumor-associated macrophages, displayed negative correlation with risk, suggesting it may also act to modulate the double-edged sword of cancer inflammatory responses (38).

Delving deeper into the mechanisms underlying the interplay between immunological and metabolic dysregulation in bone/cartilage malignancies, our study leveraged formal mediation modeling to uncover 5-hydroxyhexanoate as a key mechanistic mediator. This metabolite, generated through the intricate catabolism of amino acids and fatty acids, showed a significant specific indirect influence on the pathway linking activated HLA-DR+ NK cells to downstream changes in tumor risk. These findings suggest that SSC-A on HLA DR+ NK cells and 5-hydroxyhexanoate could serve as valuable biomarkers for the early detection and monitoring of bone and cartilage tumors. Therapeutically, strategies aimed at modulating NK cell activity and metabolic processes hold promise. For instance, inhibiting the synthesis or enhancing the metabolism of 5-hydroxyhexanoate could reduce its pro-oxidative effects, potentially slowing tumor progression. The integration of genetic instruments in our analysis has provided robust causal insights that can inform the development of targeted therapies. Future research should focus on validating these findings in diverse populations and exploring the therapeutic potential of targeting these pathways. The complex biochemical actions of 5-hydroxyhexanoate, particularly its ability to induce oxidative damage at high levels, illuminates how aberrant immune cell responses can potentially disrupt its normal homeostasis and set off a biochemical cascade fostering tumorigenesis via redox dysregulation (39, 40). The interplay between immune activation and metabolic dysregulation highlights the complexity of tumor biology and underscores the need for therapeutic strategies that address both aspects. Targeting the metabolic pathways associated with immune cell activation could mitigate the pro-tumorigenic effects of immune dysregulation and offer new avenues for treatment.

The calculated mediation ratio of 7.2% quantifies the substantial role of 5-hydroxyhexanoate as a propagator of the signalling events elicited by immune activation, although it represents one of likely multiple parallel factors in this intricate web of molecular interactions. Additional metabolites may also participate in similar fashions to 5-hydroxyhexanoate at various points along the mechanistic chain. Nevertheless, the identification of 5-hydroxyhexanoate as a contributor to this pathway provides tangible proof-of-concept of utilizing mediation modeling to dissect complex, multistep biological processes implicated in rare tumor types.

These causal findings are not merely academic; they offer tangible and actionable targets for therapeutic intervention and treatment strategies. For example, agents that modulate 5-hydroxyhexanoate availability by either inhibiting its synthesis or enhancing its further metabolism present promising approaches to mitigate its harmful pro-oxidative effects in the context of immune dysfunction (41). Furthermore, circulating 5-hydroxyhexanoate levels emerge as a potential clinically useful biomarker for non-invasive, longitudinal monitoring of personalized cancer risk, early detection of recurrence, and treatment efficacy in bone/cartilage cancer patients (42). More broadly, the approach of integrating genetic instruments to strengthen causal inference exemplified in our study should be incorporated more widely in cancer biology research to unravel mechanisms of diverse malignancies.

Some limitations of this work warrant consideration. The study population comprised predominantly individuals of European descent, necessitating additional analyses across more diverse ethnicities and ancestries (43). We were restricted to examining easily assessed circulating factors in the blood, providing an incomplete picture lacking potentially critical tissue-level and solid tumor microenvironmental data. Our genetic association findings require further experimental validation through cell line and animal model studies to definitively confirm the functional impacts of prioritized immune cell types and metabolites on bone/cartilage tumor biology. Nevertheless, our innovative methodology overcomes key confounds in conventional observational studies to elucidate causal immunometabolic pathways implicated in rare yet aggressive malignancies where traditional mechanistic studies are hampered by limited patient samples (44).

In summary, this Mendelian randomization study sheds new light on key immune cell subsets and metabolic pathways that influence the pathogenesis of rare but clinically challenging bone and cartilage cancers. The findings nominate concrete prognostic biomarkers and therapeutic candidates at both immunological and metabolic levels that could translate to improved early detection, management, and clinical outcomes for patients with these malignancies that currently lack sufficient treatment options. Moreover, the formal mediation modeling provides an analytical framework to systematically unravel step-wise causal mechanisms that can be applied to diverse tumor types beyond the scope of this study. This work exemplifies the power of capitalizing on large-scale genome-wide association study resources through genetic instrumental variable techniques to derive biologically relevant insights that can drive innovation in oncology precision medicine.

Looking ahead, follow-up investigations should expand the mediation modeling to map a more comprehensive network and identify additional players in the mechanistic chain linking immune activity to metabolic dysregulation to bone/cartilage malignancy outcomes. It will also be critical to delineate which specific stages of tumor initiation versus progression are most impacted by the prioritized cell types and metabolites. From a clinical translation perspective, development of rapid assays to monitor circulating SSC-A levels and 5-hydroxyhexanoate abundance could enable convenient liquid biopsies for early detection and treatment monitoring. Additionally, high-throughput drug screens should be undertaken to uncover agents that can selectively normalize the phenotypes of overactivated HLA-DR+ NK cells and modulate 5-hydroxyhexanoate availability. Cancer immunotherapies targeting the HLA-DR+ NK axis and drugs that interact with 5-hydroxyhexanoate-associated pathways represent particularly promising avenues for future drug development and repurposing efforts. With additional research building on the blueprint provided by our study, the long-term goal is to translate these insights into new targeted diagnostics and treatments that will tangibly improve the prognosis and quality of life for patients afflicted with rare cancers of the bone and cartilage.

Our objective in future is to move from the observational insights gleaned from genetic associations to actionable clinical interventions and diagnostics. To this end, we plan to develop a clinical research protocol that involves enrolling patients with diagnosed or suspected malignant tumors of bone and articular cartilage. We will collect plasma samples from these patients to evaluate the HLA DR+NK cell phenotype SSC-A and the levels of the metabolite 5-hydroxyhexanoate. The aims of this proposed clinical study are twofold: Validation of Biomarkers and Prospective Cohort Analysis. Additionally, given the promising role of these biomarkers, we plan to explore their potential as therapeutic targets. The interventional part of the study would involve: 1. Targeted Therapeutic Interventions: If initial validation is successful, we will explore targeted interventions that modulate the levels of these biomarkers and assess the clinical outcomes, which could lead to the development of novel therapeutic strategies. 2. Clinical Trials: Depending on the outcomes of the targeted interventions, we will design phase I/II clinical trials to test the efficacy and safety of new treatment modalities that either target the HLA DR+NK cell phenotype SSC-A directly or modulate the level of 5-hydroxyhexanoate.

This upcoming clinical study will include a diverse patient population to ensure generalizability of the findings. We aim to collaborate with multiple centers to enrich the cohort diversity and strengthen the validity of our results. The potential impact of these biomarkers on patient care and their application in the clinic could be profound, offering a new avenue for early detection, monitoring, and personalized treatment strategies for patients with malignant tumors of bone and articular cartilage.

## Data Availability

The original contributions presented in the study are included in the article/**Supplementary Material**. Further inquiries can be directed to the corresponding author.
